# Wild boar (*Sus scrofa*) carcasses as an attraction for scavengers and a potential source for soil contamination with the African swine fever virus

**DOI:** 10.3389/fvets.2024.1305643

**Published:** 2024-03-12

**Authors:** Lea Tummeleht, Susanna Suvi Siviä Häkkä, Margret Jürison, Annika Vilem, Imbi Nurmoja, Arvo Viltrop

**Affiliations:** ^1^Institute of Veterinary Medicine and Animal Sciences, Estonian University of Life Sciences, Tartu, Estonia; ^2^Institute of Agricultural and Environmental Sciences, Estonian University of Life Sciences, Tartu, Estonia; ^3^The National Centre for Laboratory Research and Risk Assessment, LABRIS, Tartu, Estonia

**Keywords:** common raven (*Corvus corax*), ASFV infection, virus DNA, ASFV p72 gene, carcass decomposition

## Abstract

The wild boar (*Sus scrofa*) is a social animal species native to Eurasia. During the last decade, the wild boar population in Estonia has been severely affected by the African swine fever virus (ASFV), which has also affected domestic pig farming. The potential transmission routes of ASFV remain unclear and are currently under intensive investigation. This pilot study aimed to clarify the frequency and characteristics of contacts between living wild boars and the carcasses of their conspecifics, which could play a role in the transmission of ASFV. Wild animals' contact and scavenging behavior on wild boar carcasses were studied using trail cameras in an experimental setting on Hiiumaa, Western Estonia. Four legally hunted carcasses were used in the present study. This study aimed to determine whether intraspecies scavenging occurs in wild boars. The persistence of ASFV DNA in soil contaminated with infected wild boar carcasses was investigated separately. Among the 17 identified wildlife species that visited wild boar carcasses, the common raven (*Corvus corax*) was the most frequent one (37.26%), followed by raccoon dogs (*Nyctereutes procyonoides*; 4.25%), carcass conspecific/wild boars (3.16%), and red foxes (*Vulpes vulpes*; 2.14%). Regarding the direct contact with the carcass, the same species ranking was detected: common raven (74.95%), raccoon dogs (9.94%), wild boars (4.21%), and red foxes (4.21%). No clear signs of cannibalism were noted among the wild boars, although brief physical contact with the carcasses was evident. The persistence of ASFV DNA in soil contaminated by infected wild boar carcasses was investigated separately. This study revealed that ASFV DNA from infected carcasses could be detected in forest soil for prolonged periods, even after removing the carcasses. Hence, the carcasses of infected wild boars may play an important role in spreading the African swine fever virus in wild boar populations; thus, prompt removal and disinfection of the soil could be considered necessary to limit the spread of the infection.

## 1 Introduction

Scavenging infected animal carcasses is considered a potential transmission route for many pathogens in wildlife (1). There are several potentially scavenging-mediated infectious diseases including tuberculosis, brucellosis, anthrax, tularemia, and African swine fever (ASF), as the causative agents can be ingested [e.g., (1–4)]. Furthermore, the opening of the carcass by vertebrate scavengers can cause pathogens (e.g., anthrax-causing bacteria *Bacillus anthracis*) to depart the carcass, contaminate the environment, or facilitate further spread by vectors (1). However, especially for birds of prey, eating carcasses helps reduce the potential of an infected source to spread infection (1). It has been shown that, in the specialized digestive system of raptors (e.g., low gastric pH and specialized microbiomes), most pathogens will not survive [e.g., (5)], and removing carcasses quickly from the environment prevents heavy pathogen loads in the substrate below the carcass (6). The other factor that reduces the infection load of carcasses is the natural decomposition process that occurs relatively rapidly. As a result, there is only limited availability of carrions for scavengers to consume (6). However, almost all carnivorous vertebrates should be regarded as facultative scavengers because they frequently contact and/or consume fresh carcasses when available (6). Hence, scavenging refers to a process that should be followed to understand disease outbreaks and reservoirs in wildlife better.

Since 2007, when African swine fever virus genotype II was first detected in Eastern Europe, wild boars (*Sus scrofa*) have played an important role in the rapid spread of the disease (7, 8), which was also observed in Estonia. Wild boars have experienced re-emerging outbreaks since the introduction of the African swine fever virus (ASFV) in Estonia in September 2014 (9, 10). Intraspecies scavenging among wild boars has been suggested as a possible means of transmission of ASFV; however, the scavenging behavior of wild boars has not been extensively studied, and it seems that wild boar behavior differs between countries (11–13). As social animals, suids do not always scavenge but investigate their deceased conspecifics and use their natural rooting behavior to search for the soil under the carcass [e.g., (12)]. Physical contact with pathogen-positive carcasses or the substrates beneath them poses an equal risk of ASFV infection. A large-scale study in Germany showed that ~30% of the encounters of wild boars with dead conspecifics led to direct contact: sniffing and poking on the carcasses, whereby animals were particularly interested in soil under and around the carcasses (12). We did not observe any evidence of intraspecific scavenging. Regarding contact with wild boar carcasses, either via hunting or otherwise, it is recognized that humans are the main contributors to virus transmission and virus introduction into domestic pig farms (14).

Recent studies have concluded that ASFV exhibits exceptional environmental stability and resilience (15). Infected tissues and organs from decomposing carcasses that persist in the environment for a long time can be the sources of ASFV infection for several months, particularly at low temperatures (15, 16).

The general objective of this pilot study was to discuss the scavenging behavior of Estonian wildlife on wild boar carcasses. The study specifically focused was on wild boar behavior to detect cannibalism or other types of physical contact with dead conspecifics to understand the role of wild boar behavior in spreading ASFV within the population. Additionally, in a separate experiment, the resilience of the ASF viral DNA in the soil under the infected carcass was tested over time.

## 2 Materials and methods

### 2.1 Study design

#### 2.1.1 Study 1. Investigating wild boar behavior concerning a conspecific carcass

The field experiment was conducted in a forest on the Hiiumaa island (58°53′ 3″N, 22°38′40″ E), the second largest island in Estonia, located in the Baltic Sea, which is 22 km west of the mainland.

This location was selected for four reasons: First, to date, no cases of ASF were detected in Hiiumaa, and the experimental work did not interfere with the infectious status of the island. Second, since there was a legal obligation to remove all wild boar carcasses found in the forest in infected areas by hunters in Estonia, other locations were not qualified for this study. Third, ASF was a highly lethal disease, especially when first discovered, so there were not enough wild boars in study areas where ASFV spreads. The holders of the hunting grounds agreed to deliver the hunted carcasses. Being able to buy the carcasses was highly valuable as, at that time, most of the wild boar population of the country had been decreased by the disease, and the hunters were not interested in discarding the hunted game. Fourth, the hunting grounds were easily accessible.

Four legally hunted wild boar carcasses were purchased from local hunters. All carcasses tested negative for ASFV at the Estonian National Center for Laboratory Research and Risk Assessment (LABRIS; Tartu, Estonia; https://labris.agri.ee/en). For carcass testing, serum samples were collected and analyzed by real-time PCR using ASFV p72 gene-targeting forward and reverse primers and TaqMan probes, as mentioned in the study conducted by Tignon et al. (17).

Table 1 shows the details of the carcasses used in this study. No animals were killed during this study.

**Table 1:** Parameters and persistence of carcasses used for the scavenging experiment.

**Carcass ID**	**Sex**	**Age (years)**	**Weight (kg)**	**Date of placing**	**Date of only bones and skin left**	**Persistence of carcass in days**	**Remarks**
Carcass 1	Female	2+	90	21.11.2016	26.12.2016	35	
Carcass 2	Male	4	70	11.01.2017	29.01.2017	18	
Carcass 3	Male	4–5	100	13.02.2017	27.03.2017	42	
Carcass 4	Male	2+	60	06.8.2017	14.08.2017	8	Head removed; gut pile placed aside

The animals were monitored using the UOVision (Shenzhen, China) trail camera, which is a UM595-2G model (infrared heat and motion-sensitive wireless digital devices). The monitoring period was from 21.11.2016 to 18.10.2017, which lasted for 332 days. Two cameras were set to simultaneously focus on the carcasses (in the case of carcass 4, besides the gut pile) from different directions. The cameras were fixed on trees 5 m from the placed carcass and at a height of 1.5 m above the ground. The cameras were programmed to take three photos when activated by animal movements, with a 1-min pause between every subsequent activation.

**Figure 1:**
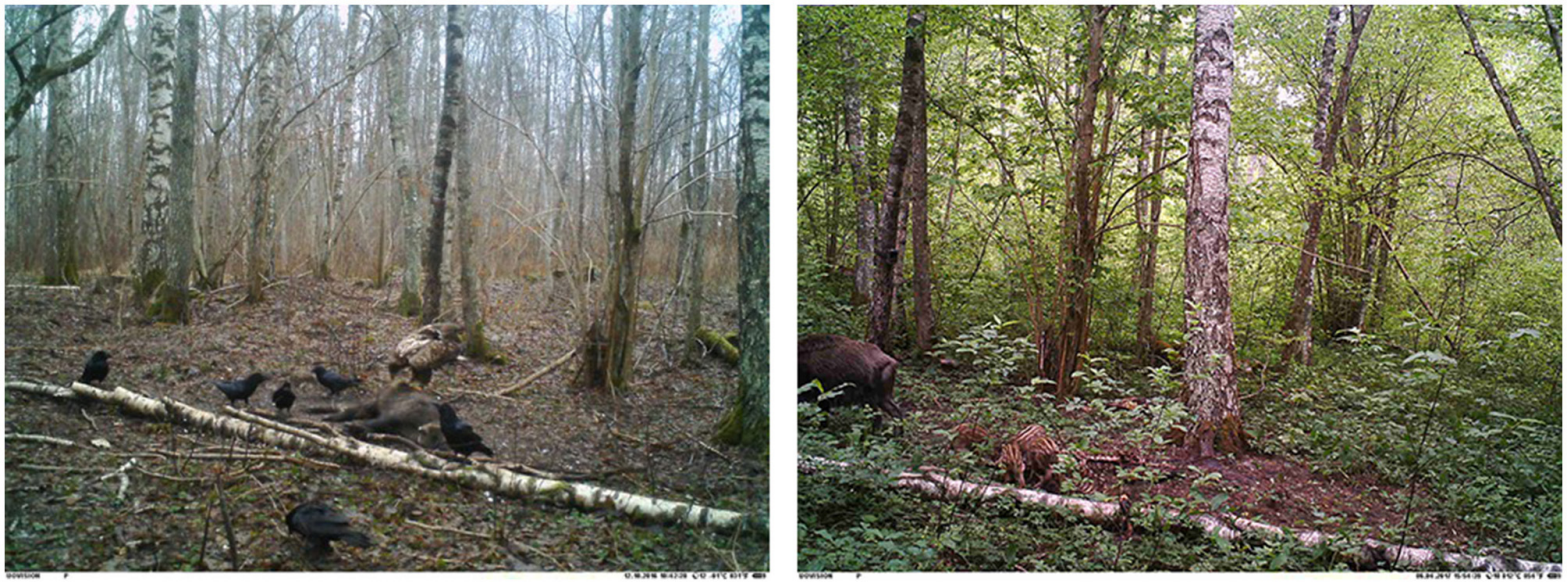
Trail camera photographs of random visitors of experimentally placed wild boar carcasses in the study area—forest on the island Hiiumaa.

One of the cameras stopped working a couple of times and did not film during the following periods: 26 November 2016–13 December 2016, 25 January 2017–14 April 2017, and 8 June 2017–25 July 2017. In total, 16,967 individual pictures from the two cameras were collected and included in the analysis. Examples of the trail camera photographs are shown in Figure 1.

#### 2.1.2 Study 2. Estimating the persistence of soil contamination under the ASFV-infected wild boar carcass

To estimate how long the ASFV DNA released from infected wild boar carcasses was detectable, soil samples were collected from the carcasses of infected dead wild boars (infection confirmed at the LABRIS) after removing the carcasses. In total, between 2016 and 2017, samples were collected from 11 sites in four Estonian counties (Järvamaa, Jõgevamaa, Lääne-Virumaa, and Raplamaa) where wild boar carcasses were found. In total, 107 soil samples were collected at ~1–2 week intervals.

In July 2016, a pilot study was conducted to optimize the methodology by sampling the soil from three nearby ASFV-infected carcasses. Samples were manually collected from the surface of each site using a latex glove. A handful of soil was taken, and the glove was pulled from the hand around the sample, tied, placed in a sealable transportation container, and transported to the laboratory within 24 h. Samples were collected for 2–4 weeks (see Supplementary Table 1), with two samples per site at every sampling event.

Sampling in October 2016 and February 2017 was performed using a sampling tool consisting of a 50 ml plastic syringe, the top of which was cut off. The syringe was pushed into the soil, and the sample stuck in a barrel (up to 50 ml) was pushed into a sample container using a plunger. Separate syringes were used at each sampling site. Three samples were collected underneath each carcass at every sampling point and were transported to the laboratory within 24 h. All samples were stored at −20°C until further analysis. The samples were collected from the carcasses discovered in October over 1 month, unless the sampling site was destroyed by plowing or flooding (see Supplementary Table 1). From the carcasses discovered in February, samples were collected for 4 months, assuming a longer survival time for the virus in winter.

Viral DNA analyses were performed periodically after collecting samples from several sites, rather than immediately after sampling. According to the manufacturer's guidelines, total viral DNA was isolated from the soil samples using the PowerSoil DNA Isolation Kit (MO BIO Laboratories, Carlsbad, California, United States). The DNA samples were analyzed by real-time PCR using ASFV p72 gene-targeting forward and reverse primers and TaqMan probes, as detailed by Tignon et al. (17). Samples were considered negative when there was no threshold cycle (Ct value) or the Ct value was >40 in the PCR analysis.

Virus DNA-positive soil samples were investigated at the Friedrich-Loeffler Institute (Germany) for the presence of viable viruses. No virus was isolated from cell cultures (data not shown).

### 2.2 Data analysis

Each photo was visually assessed by one author (S.H.) to determine the animal species in the photo and to record the scavenging behavior of the animal (such as tearing, removing, chewing, or breaking down soft tissues and bones). When additional assistance was required, a wildlife veterinarian (M.L.) was consulted to determine the species in the photograph. The data from the trail camera recordings based on the information in the pictures were collected using an MS Excel spreadsheet. The variables recorded were as follows: date of the event; the beginning of the event when the animal/animals were first seen on camera; end of the event when the animal/animals were last seen on camera; species; the number of animals of the same species in an event; camera ID; air temperature (°C); detection of contact with the carcass (yes/no); the number of animals in contact during the event, and extra notes when necessary.

The collected data were analyzed, and graphs were constructed using MS Excel.

## 3 Results

### 3.1 Animals visiting wild boar carcasses

From the camera recordings, 17 vertebrate species, 10 mammals, and 7 avian species roamed around the experimentally placed wild boar carcasses; there were 4,337 individual encounters. Most observed encounters were birds (79.59%): common raven (*Corvus corax*), common buzzard (*Buteo buteo*), common crane (*Grus grus*), Eurasian magpie (*Pica pica*), hooded crow (*Corvus cornix*), golden eagle (*Aquila chrysaetos*), and white-tailed eagle (*Haliaeetus albicilla)*. Among all the recorded animals belonging to mammalian species (20.41%), the distribution is as follows: raccoon dog (*Nyctereutes procyonoides*), wild boar (*Sus scrofa*), red fox (*Vulpes vulpes*), gray wolf (*Canis lupus*), European pine marten (*Martes martes*), roe deer (*Capreolus capreolus*), red deer (*Cervus elaphus*), European elk (*Alces alces*), and domestic cat (*Felis catus*). Additionally, unidentified mammalian and avian species were grouped. Common ravens were the most frequently identified (*n* = 3,231), followed by raccoon dogs (*n* = 369), wild boars (*n* = 268), and red foxes (*n* = 186).

A total of 2,303 encounters from 11 identified species and two unidentified mammal species were found to have direct contact with wild boar carcasses (Tables 2, 3). All species in contact, excluding wild boars and Eurasian magpies, were also found scavenging on the carcass. Peaks in animals detected and in contact occurred 1 week or less, before only bones and skin were left from the carcass. Common ravens were the most common species in contact with wild boar carcasses, followed by raccoon dogs. The third highest frequency rank of contact with the carcass was shared between the red fox and wild boar.

**Table 2:** Number and percentage of individual animals of different species in contact with the carcasses.

**Species**	**Number of individuals in contact**	**Percentage of all individuals in contact**
Common raven (*Corvus corax*)	1,726	74.95%
Raccoon dog (*Nyctereutes procyonoides*)	229	9.94%
Wild boar (*Sus scrofa*)	97	4.21%
Red fox (*Vulpes vulpes*)	97	4.21%
Common buzzard (*Buteo buteo*)	63	2.74%
White-tailed eagle (*Haliaeetus albicilla)*	33	1.43%
Hooded crow (*Corvus cornix*)	22	0.96%
Gray wolf (*Canis lupus*)	15	0.65%
Eurasian magpie (*Pica pica*)	8	0.35%
Golden eagle (*Aquila chrysaetos*)	6	0.26%
European pine marten (*Martes martes*)	5	0.22%
Unidentified mammal	2	0.09%
Total number of individuals in contact	2,303	100%

**Table 3:** Characteristics of wild boar visits to the carcasses.

**Carcass ID**	**Date of placing**	**Date of only bones and skin left**	**Total no of wild boar visited (in contact)**	**First wild boar visits to the carcass**	**First wild boar contacts with the carcass (description)**
Carcass 1	21.11.2016	26.12.2016	41 (11)	22.11.2016	03.12.2016 (piglets, adult avoiding the carcass)
Carcass 2	11.01.2017	29.1.2017	20 (10)	11.01.2017	26.01.2017 (one adult); 30.01.2017 (eight animals, mother and piglets)
Carcass 3	13.02.2017	27.3.2017	150 (74)	15.02.2017	26.03.2017 (two adults)
Carcass 4	06.08.2017	14.8.2017	57 (3)	08.08.2017	8.08.2017 (one adult)

The observational period of one carcass is counted from the date of placing this carcass until the day next carcass was placed.

The cameras recorded a total of 268 wild boar visits (the total number of counted wild boars in photos from one visitation event) to the site where carcasses were placed (which accounted for 6.18% of the total number of all animal encounters), while 97 of these visits (33.92%) were instances where wild boars came into direct contact with the carcasses. The period from the placement of the carcass to the first contact with the wild boar varied between 0 and 2 days. The availability of soft tissues from the carcasses (excluding the skin) varied from ~7–44 days (Table 3). The shortest wild boar contact lasted 1 s, the longest was 22 min 32 s, and the median value of boar contacts was 4 min 30 s. However, there were no clear signs of intraspecies scavenging in wild boars. Piglets or their mothers were in contact with several wild boars. In addition, piglets were often observed when no contact was recorded.

**Figure 2:**
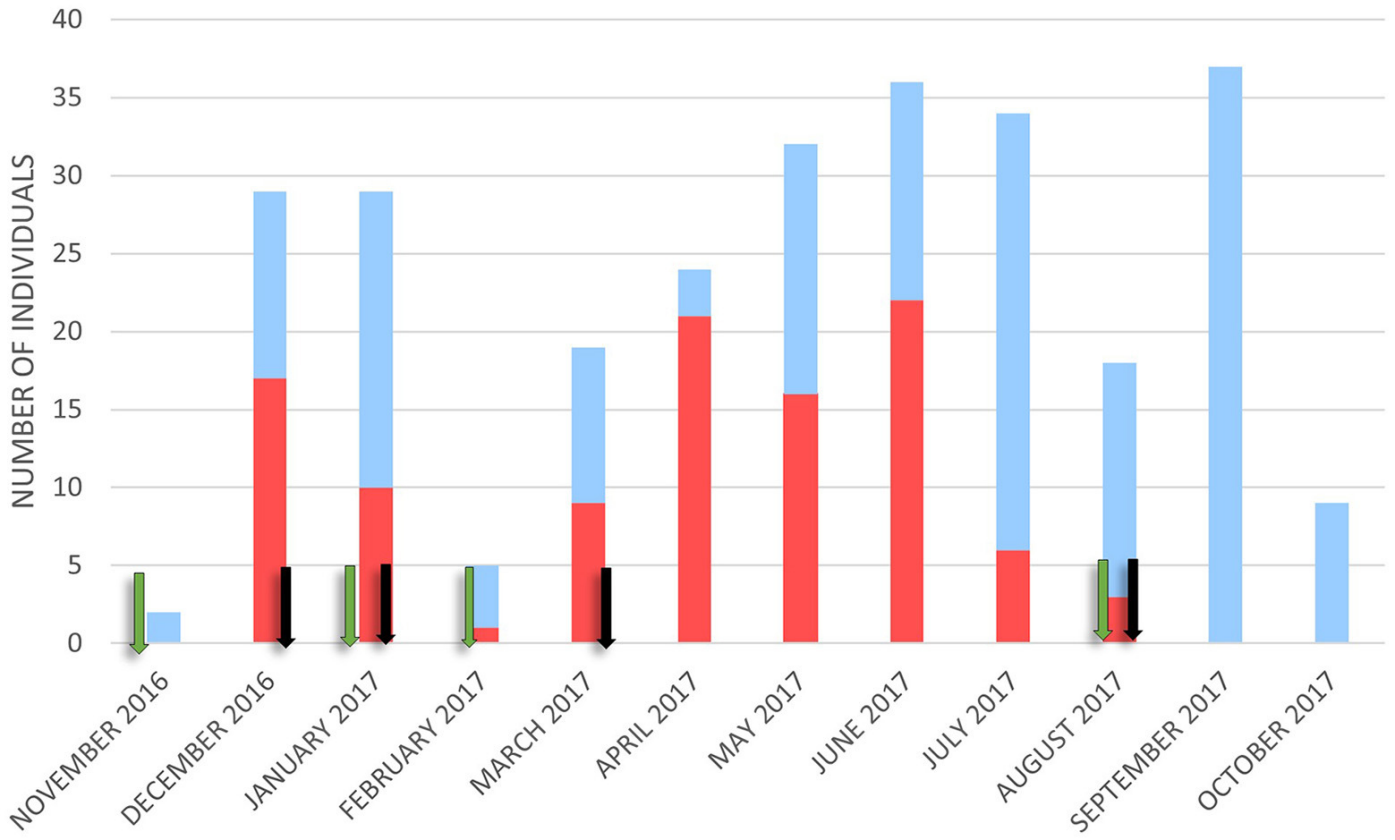
The number of wild boars was detected in contact (red bar) and not in contact (blue bar) with the carcasses monthly. Green arrows represent when new carcasses were placed and black arrows indicate when only bones and the skin were left.

Wild boar visits were recorded throughout the year, with the lowest number in November (*n* = 2) and the highest in September (*n* = 37). Most contacts with carcasses occurred in June (*n* = 22) and April (*n* = 21). No contact was observed between September and November (Figure 2).

### 3.2 ASFV soil contamination persistence in time

Of the 11 collection sites in the two individual sites, the soil samples were negative from the first sampling event. At one of these sites, the removed carcass was an ~1.5-month-old skeleton. In the other case, there was a fresh carcass that was 1–2 days old.

The first soil samples were ASFV DNA-positive at the nine sampling sites, whereas the removed carcass characteristics varied greatly (Table 4). There were carcasses ranging from 1–2 days old to 2–3 weeks old, some of which were intact while others had already been eaten by wild animals. Natural decomposition was already in progress in some cases. ASFV DNA was detectable in average after 28 ± 24 (mean ± SD) days after the first sampling event. The site with the longest detectable ASFV DNA presence was after 82 days (from 17 February 2017 to 10 May 2017), and the shortest period was <7 days (10 October 2016 positive; after a week, faded). The carcass conditions are shown in Table 4.

**Table 4:** African swine fever virus (ASFV) positive samples collected in the site of the removed infected wild boar carcasses.

**ID of carcass**	**Month of discovery**	**No of samples**	**No of positive samples**	**% Of positive samples**	**No of days virus detectable**	**Conditions of the carcass when found**
V1	July	6	4	66.7%	15	Adult female. Fresh carcass of 1–2 days. Located in open area exposed to the sun.
V2	July	4	0	0.0%	0	Adult female. Fresh carcass of 1–2 days. Located under the trees in shadow.
V3	July	4	1	25.0%	7	Adult female. Carcass of approximately a week old; decomposed. Located under the trees in shadow.
V4	July	8	0	0.0%	0	Approximately 1.5 months old carcass of only bones left.
H1	October	12	8	66.7%	43	Fresh carcass of 1–2 days. No signs of hemorrhage.
O1	October	9	2	22.2%	15	Fresh carcass of about 1 day. Symptoms of ASF: bleeding eyes and gums; bruises on the mucous membrane, reddened groin and armpits.
SO1	October	4	1	25.0%	1	Fresh carcass of about 2–3 days. The scavengers have eaten the head and behind. No signs of hemorrhage.
SA1	October	9	3	33.3%	19	Fresh carcass of about 2–3 days. The scavengers have eaten the behind. Symptoms of ASF: bleeding eyes and gums; bruises on the mucous membrane, reddened groin and armpits.
VI1	October	9	5	55.6%	34	Fresh carcass of about 3–4 days. Scavengers have eaten the behind. Bruises in the armpits, eyes slimy.
US1	February	21	9	42.9%	82	Skeleton; age > 1 month.
US2	February	21	14	66.7%	82	Carcass of 2–3 weeks; half body eaten by scavenger; the rest of the remains frozen.

## 4 Discussion

### 4.1 Animals visiting wild boar carcasses

Vertebrate animals, 4,337 individual encounters, were detected by trail cameras to visit the wild boar carcasses in this study belonged to 10 different mammal species and seven avian species. Furthermore, avian visits were more frequent. The ranking of the animal species most frequently identified and in physical contact with the carcass fully coincided as follows: common raven, raccoon dog, wild boar, and red fox. The most common raven carcass contact was detected during winter (January and December). Similarly, Probst et al. (4) used wild boar carcasses to collect data on vertebrate scavengers in northeast Germany. Their analysis revealed that birds were the first to detect carcasses in winter/spring or forest clearings when the data were analyzed separately by seasons and site visibility. However, in the summer/autumn and closed forests, mammals were first found at the site of the carcass (4). Unfortunately, in the current study, we did not have enough carcasses for statistical power to investigate the species abundance between seasons.

Among mammalian species, raccoon dogs and red foxes were the most frequently expected visitors around the carcasses in the forest in Hiiumaa. Similarly, the study by Probst et al. (4), performed in northeast Germany, found that raccoon dogs were the most frequent (44% of the observations) mammal species visiting the wild boar carcasses. Raccoon dogs and red foxes were scavenging on wild boar carcasses. In addition, six other mammalian species were considered as potential scavengers, i.e., wild boars, raccoons (*Procyon lotor*), martens (*Martes sp*.), polecats (*Mustela putorius*), water voles (*Arvicola terrestris*), and domestic dogs (*Canis familiaris*) (4). A comprehensive study of 214 naturally occurring carcasses in Poland also ranked red foxes and raccoon dogs as the most frequently scavenged mammals in temperate European woodlands (18). Although the overall composition of the fauna is different in central Spain, a study by Carrasco-Garcia et al. (2) showed that red foxes were among the most frequent scavengers, followed by griffon vultures (*Gyps fulvus)*, and common ravens.

The first contact of the wild boar with their conspecific carcass occurred 2 days after the carcass placement. Wild boars were most often detected to root in the remains of carcasses, bones, and skin. A similar behavioral trend has been observed in Germany (4).

Although wild boars were in direct contact with the carcass, none of them were detected in the acts of cannibalism. Direct contact with the carcasses was not frequent and usually of short duration, lasting 1–3 min. Although no direct eating of carcasses was observed, the contact with carcasses can be assumed to be intense enough for virus transmission to occur if the infectious virus was present in the carcass or the contaminated soil around it. Unfortunately, no quantification supporting this hypothesis was found in the scientific literature. Studies conducted in experimental settings have demonstrated that contact with an environment contaminated with ASFV can lead to infection in susceptible pigs (19).

The lack of signs of cannibalism in our pilot study differed from those reported in other European countries [e.g., (11, 13)]. As our study was conducted on an island and at one location, it represents the behavior of a limited metapopulation of wild boars in Estonia. Therefore, it is impossible to extrapolate these findings to the entire population of a country. Nevertheless, these findings coincide with the results of a much larger study conducted by Probst et al. (12) conducted in Northern Germany, where wild boar scavenging on red deer (*Cervus elaphus*) and roe deer (*Capreolus capreolus*) carcasses was observed; however, there were no clear signs of intraspecies scavenging, even when direct contact was recorded (12). Differently, one study in the Czech Republic with placed carcasses found wild boar contact with the carcass in 81% of the records. Contrastingly, in this study, cannibalism was observed in 9.8% of all recorded wild boar visits (13). In the study of wild boars in Poland, Merta et al. (11) reported that their stomach contents consisted of other animal tissues and residues of conspecifics hinting at scavenging wild boar carcasses. Nevertheless, since wild boars are omnivorous animals exhibiting strong rooting behavior, even when not scavenging, they significantly impact the surfaces on which they forage (20). Hence, deliberate or accidental consumption of carcasses or invasive contact with carcasses can be foreseen.

Cannibalistic behavior may be related to the availability of resources for wild boars. In the northern latitudes, the density of wild boars is generally lower than that in the southern latitudes [e.g., (21)]. On the one hand, this is due to the difference in available feeding resources. Still, on the other hand, a higher density means more competition for resources between animals, which may lead to different animal behaviors (22). We may also speculate that wild boars in Southern and Central Europe may have more hybrids with domestic pigs, whereas, in the northern latitudes, wild boars seem to be of pure wild boar genotype (23). Since cannibalism is common in domestic pigs and may have a genetic background, northern wild boars might have less cannibalism. However, this hypothesis requires further investigation. A study conducted in Germany, to assess wild boar behavior concerning dead conspecifics, observed wild boars scavenging red deer (*Cervus elaphus*) and roe deer (*Capreolus capreolus*) carcasses. Still, there were no clear signs of intraspecies scavenging even when direct contact was recorded (12).

However, we observed many wild boar contacts with the remains of the carcasses (bones and the skin) and rooting of the soil around the carcasses; this could indicate that the sites of carcass decomposition remain attractive for wild boars for extended periods and may serve as hubs for virus transmission, as long as the virus persists in the carcass or the surrounding soil. Wild boars' natural behavior of rooting, wallowing, and investigating objects can be a risk factor for acquiring the infection when living in a virus-contaminated environment. Pepin et al. (24) developed a model (Bayesian computation) of ASFV in wild boars to estimate the virus transmission via carcasses in Eastern European wild boars. They inferred 53–66% of carcass-based transmission events when live conspecifics were in contact.

### 4.2 The persistence of ASFV DNA in the soil contaminated with infected wild boar carcasses

Infected carcasses are a potential source of environmental contamination, particularly the soil under the carcass and other objects around [e.g., (25, 26)]. The results of soil contamination testing in this study showed that viral DNA was detectable in soil contaminated with ASFV-infected carcasses, long after the carcasses were removed. Viral DNA contamination in the soil was detectable on average 1 month after carcass removal, independent of the season. When planning the current pilot study, we based the preliminary knowledge on half-lives of ASFV DNA in tissue samples stored at 20°C from 1.7 to 7.4 days (27). Hence, the sampling period was ~2 months. Therefore, real DNA persistence times often exceed the planned sampling periods.

Although we could not detect the total disappearance of DNA in the soil at every study site, the sites of ASFV-infected carcasses seemed to show the longest detectable viral DNA traces discovered in winter (February), followed by the sites discovered in autumn (October). The shortest persistence of viral DNA was observed in samples from sites detected in July. We could not test whether ambient temperature and season effects were statistically significant due to insufficient data. However, it is generally established that ASFV persists longer at low temperatures, particularly in raw meat products [e.g., reviewed by Chenais et al. (14)]. Under laboratory conditions, a study investigated the soil samples and tested the survival of ASFV under different temperatures. The virus was detectable at day 112 when stored at 4°C, and the genome copy numbers were constant over 210 days after soil inoculation, showing a clear temperature dependency (28). Often, the persistence of ASFV in soil is determined by parameters other than temperature, such as soil pH. For example, in acidic soil, the virus disappears more quickly (29).

The presence of viral DNA in soil does not directly indicate that the soil is infectious. However, this proves that the virus reaches the soil from the carcasses; therefore, the soil is also a potential source of infection. A study in Lithuania (30) tested the persistence of ASFV in buried ASFV-infected wild boar carcasses using *in vitro* assays and viral DNA. At most sites, excavated carcass samples were positive for the ASFV genome, whereas no livable ASFV was isolated from any of the carcasses. Similarly, a study conducted in the Tavush region of Armenia investigated the presence of ASFV DNA in bone, bone marrow, and porcine tissue samples obtained from skeletons and carcasses found in forests and excavated from cemeteries; however, the study found that infectious ASFV could not be isolated (31).

It is important to conclude that, although direct contact between wild boars and the carcasses of their conspecifics was not frequent, the rooting behavior and intensive investigation of the remains of young pigs should be considered as critical factors in the chain of disease transmission. Therefore, it is crucial to quickly remove ASFV-infected carcasses from the environment. Furthermore, directly causing infection to the visiting wild boar, the carcass can also provide a basis for the virus to be carried by potential arthropod vectors. ASFV DNA was detected in the soil at the carcass removal site for weeks. Although the potential for live virus has not been studied, infected carcasses not removed from the site could increase the risk of indirect virus spread.

## Data availability statement

The original contributions presented in the study are included in the article/Supplementary material, further inquiries can be directed to the corresponding author.

## Ethics statement

Ethical approval was not required for the study involving animals in accordance with the local legislation and institutional requirements because, the wild boar carcasses were legally hunted. No animals were killed for the purpose of this study. Animals filmed by cameras were not disturbed nor harmed during the study.

## Author contributions

LT: Conceptualization, Data curation, Formal analysis, Investigation, Supervision, Visualization, Writing—original draft, Writing—review & editing. SH: Conceptualization, Data curation, Formal analysis, Investigation, Visualization, Writing—original draft. MJ: Data curation, Formal analysis, Investigation, Writing—review & editing. AVile: Data curation, Formal analysis, Writing—review & editing. IN: Formal analysis, Project administration, Writing—review & editing. AVilt: Conceptualization, Data curation, Funding acquisition, Project administration, Resources, Supervision, Writing—original draft, Writing—review & editing.
