# Supplementary material for: Wild boar (Sus scrofa) carcasses as an attraction for scavengers and a potential source for soil contamination with the African swine fever virus
Source: Front Vet Sci. 2024 Mar 12;11:1305643. doi: 10.3389/fvets.2024.1305643 (PMC10967021; doi:10.3389/fvets.2024.1305643)
Supplement: Supplementary file 1 [file Table_1.docx]

**Supplementary table 1.** Characterization of the wild boar carcasses and sampling the soil under African swine fever virus (ASFV) infected carcasses that were moved after discovering.

| **Sampling place and events** | **No of samples** | **No of positive samples** | **Conditions of the carcass when found** |
| --- | --- | --- | --- |
| V1 total no of samples: | 6 | 4 | Adult female wild boar. Fresh carcass of 1-2 days. Located in open area exposed to the sun.  ASFV DNA detectable for 15 days. |
| 1.07.2016 | 2 | 2 |  |
| 8.07.2016 | 2 | 1 |  |
| 16.07.2016 | 2 | 1 |  |
| V2 total no of samples: | 4 | 0 | Adult female wild boar. Fresh carcass of 1-2 days. Located under the trees in shadow.  ASFV DNA detectable for 0 days. |
| 1.07.2016 | 2 | 0 |  |
| 8.07.2016 | 2 | 0 |  |
| V3 total no of samples: | 4 | 1 | Adult female wild boar. Carcass of approximately a week old; decomposed. Located under the trees in shadow.  ASFV DNA detectable for 7 days. |
| 1.07.2016 | 2 | 0 |  |
| 8.07.2016 | 2 | 1 |  |
| V4 total no of samples: | 8 | 0 | Approximately 1.5 months old wild boar carcass of only bones left.  ASFV DNA detectable for 0 days. |
| 29.07.2016 | 2 | 0 |  |
| 5.08.2016 | 2 | 0 |  |
| 11.08.2016 | 2 | 0 |  |
| 19.08.2016 | 2 | 0 |  |
| H1 total no of samples: | 12 | 8 | Fresh wild boar carcass of 1-2 days. No signs of hemorrhage.  ASFV DNA detectable for 43 days. |
| 10.10.2016 | 3 | 3 |  |
| 19.10.2016 | 3 | 2 |  |
| 31.10.2016 | 3 | 2 |  |
| 22.11.2016 | 3 | 1 |  |
| O1 total no of samples: | 9 | 2 | Fresh wild boar carcass of about 1 day. Symptoms of ASF: bleeding eyes and gums; bruises on the mucous membrane, reddened groin and armpits.  ASFV DNA detectable for 15 days. |
| 4.10.2016 | 3 | 1 |  |
| 19.10.2016 | 3 | 1 |  |
| 31.10.2016 | 3 | 0 |  |
| SO1 total no of samples: | 4 | 1 | Fresh wild boar carcass of about 2-3 days. The scavengers have eaten the head and behind. No signs of hemorrhage. Since this was on the field, it was ploughed after second sampling events.  ASFV DNA detectable for 1 day. |
| 10.10.2016 | 3 | 1 |  |
| 19.10.2016 | 1 | 0 |  |
| SA1 total no of samples: | 9 | 5 | Fresh wild boar carcass of about 2-3 days. The scavengers have eaten the behind. Symptoms of ASF: bleeding eyes and gums; bruises on the mucous membrane, reddened groin and armpits. The ditch where carcass found was later filled with rain water, so further sampling was impossible.  ASFV DNA detectable for 19 days. |
| 12.10.2016 | 3 | 2 |  |
| 21.10.2016 | 3 | 2 |  |
| 31.10.2016 | 3 | 1 |  |
| VI1 total no of samples: | 9 | 5 | Fresh wild boar carcass of about 3-4 days. Scavengers have eaten the behind. Bruises in the armpits, eyes slimy.  ASFV DNA detectable for 34 days. |
| 19.10.2016 | 3 | 2 |  |
| 31.10.2016 | 3 | 2 |  |
| 22.11.2016 | 3 | 1 |  |
| US1total no of samples: | 21 | 7 | Wild boar skeleton; age > 1 month.  ASFV DNA detectable for 37 days. |
| 8.02.2017 | 3 | 3 |  |
| 22.02.2017 | 3 | 3 |  |
| 17.03.2017 | 3 | 1 |  |
| 6.04.2017 | 3 | 0 |  |
| 26.04.2017 | 3 | 0 |  |
| 10.05.2017 | 3 | 0 |  |
| 30.05.2017 | 3 | 0 |  |
| US2 total no of samples: | 21 | 12 | Wild boar carcass of 2-3 weeks; half body eaten by scavenger; the rest of the remains frozen. Sampling of frozen soil was difficult in February.  ASFV DNA detectable for 91 days. |
| 8.02.2017 | 3 | 0 |  |
| 17.02.2017 | 1 | 1 |  |
| 22.02.2017 | 3 | 1 |  |
| 17.03.2017 | 2 | 2 |  |
| 6.04.2017 | 3 | 3 |  |
| 26.04.2017 | 3 | 2 |  |
| 10.05.2017 | 3 | 3 |  |
| 30.05.2017 | 3 | 0 |  |
| In total | 107 | 45 |  |
